# Do Not Tell People They Look Ill

**Published:** 1898-02

**Authors:** 


**Do Not Tell People They Look Ill**—In the May number of your journal, in the paragraph, "Do Not Tell People They Look Ill," I was greatly interested. It is what every one should thoroughly understand and never forget. Many sick persons that I have called on have told me that I did them more good than the medicine they were taking. I understood it, for I told them pleasant and agreeable stories, set them to laughing, and made them forget themselves, and they wanted me to call often. I could relate some wonderful experiences, but must not do it now, but I would like to emphasize your suggestion, do not tell people they look ill.—*Journal of Hygiene.*

---

**Mueller's Tooth Wash,** according to the *Centralblatt fur die gesammte Therapie* for October, is composed as follows:

| | | |
|---|---|---|
| ℞ | Thymol | 1 part |
| | Benzoic acid | 12 parts |
| | Tincture of eucalyptus | 60 parts |
| | Alcohol | 400 parts |

M. S.: A teaspoonful to be diluted with half a wineglassful of water.

